# Pharmacokinetic and Pharmacodynamic Characterisation of an Anti-Mouse TNF Receptor 1 Domain Antibody Formatted for In Vivo Half-Life Extension

**DOI:** 10.1371/journal.pone.0137065

**Published:** 2015-09-09

**Authors:** Laura J. Goodall, Milan Ovecka, Daniel Rycroft, Sarah L. Friel, Andrew Sanderson, Prafull Mistry, Marie L. Davies, A. Allart Stoop

**Affiliations:** 1 Biopharm Innovation Unit, Biopharm R&D, GlaxoSmithKline, Stevenage, United Kingdom; 2 R&D Projects, Clinical Platforms and Sciences, GlaxoSmithKline, Stevenage, United Kingdom; Universitatsklinikum Freiburg, GERMANY

## Abstract

Tumour Necrosis Factor-α (TNF-α) inhibition has been transformational in the treatment of patients with inflammatory disease, e.g. rheumatoid arthritis. Intriguingly, TNF-α signals through two receptors, TNFR1 and TNFR2, which have been associated with detrimental inflammatory and beneficial immune-regulatory processes, respectively. To investigate if selective TNFR1 inhibition might provide benefits over pan TNF-α inhibition, tools to investigate the potential impact of pharmacological intervention are needed. Receptor-deficient mice have been very insightful, but are not reversible and could distort receptor cross-talk, while inhibitory anti-TNFR1 monoclonal antibodies have a propensity to induce receptor agonism. Therefore, we set out to characterise a monovalent anti-TNFR1 domain antibody (dAb) formatted for *in vivo* use. The mouse TNFR1 antagonist (DMS5540) is a genetic fusion product of an anti-TNFR1 dAb with an albumin-binding dAb (AlbudAb). It bound mouse TNFR1, but not human TNFR1, and was an antagonist of TNF-α-mediated cytotoxicity in a L929 cell assay. Surprisingly, the dAb did not compete with TNF-α for TNFR1-binding. This was supported by additional data showing the anti-TNFR1 epitope mapped to a single residue in the first domain of TNFR1. Pharmacokinetic studies of DMS5540 in mice over three doses (0.1, 1.0 and 10 mg/kg) confirmed extended *in vivo* half-life, mediated by the AlbudAb, and demonstrated non-linear clearance of DMS5540. Target engagement was further confirmed by dose-dependent increases in total soluble TNFR1 levels. Functional *in vivo* activity was demonstrated in a mouse challenge study, where DMS5540 provided dose-dependent inhibition of serum IL-6 increases in response to bolus mouse TNF-α injections. Hence, DMS5540 is a potent mouse TNFR1 antagonist with *in vivo* pharmacokinetic and pharmacodynamic properties compatible with use in pre-clinical disease models and could provide a useful tool to dissect the individual contributions of TNFR1 and TNFR2 in homeostasis and disease.

## Introduction

TNF-α is a pleiotropic cytokine associated with both inflammatory and immuno-regulatory activities [1,2]. Its relevance to disease is well established and treatment with TNF-α antagonists has been highly efficacious in a range of inflammatory disorders, e.g. rheumatoid arthritis [3]. From a biological perspective, TNF-α mediates its effects by signalling through two distinct, specific, high-affinity receptors [4,5]. TNFR1 is expressed ubiquitously and signals through an intracellular death domain (DD), inducing apoptosis and NF-κB mediated inflammation [6]. In contrast, TNFR2 is expressed on a restricted subset of cells, including endothelial cells and cells of the immune system (T-cells) [7,8], has a TNF receptor-associated factor (TRAF) signalling domain, and has been associated with Akt/PKB-mediated repair and migration [9]. Both TNF receptors signal as membrane-anchored receptors and their numbers are regulated through a combination of receptor synthesis, internalisation and shedding, resulting in circulating soluble TNFR1 and TNFR2 [10]. As the majority of detrimental effects seem to be mediated by TNFR1 and the more beneficial processes by TNFR2, further improvements in TNF-α antagonistic therapies might be made by selectively targeting TNFR1.

Although the TNF receptors were identified and characterised nearly 30 years ago [11], the understanding of the exact roles of both receptors and their cross-talk remains unclear. Whereas TNFR1 signalling has been characterised in detail, TNFR2 signalling is less well understood as is its physiological role during disease and recovery. In part this may be due to the requirement for membrane-bound TNF-α to initiate TNFR2 signalling [12] and the absence of generally accepted intracellular markers of TNFR2 signalling. Both of these aspects complicate *in vitro* studies of TNFR2 function. In addition, the tools available to investigate the individual contributions of TNFR1 and TNFR2 cross-talk are limited. The largest contribution to our knowledge of the role of individual receptors has been made using the receptor-specific knock-out mice [13–15]. Although these mouse models have been and continue to be very insightful, they lack the ability to investigate cross-talk between receptors and would not be able to mimic the effects achieved through reversible inhibition as observed during pharmacological intervention. To provide a more pharmacologically relevant model of target inhibition, monoclonal antibodies are widely used in pre-clinical models. However in the case of TNFR1, monoclonal antibodies have been of limited *in vivo* use as inhibitory antibodies. For when inhibiting binding of TNF-α to its receptor, they have been shown to induce TNFR1 agonism through a mechanism of antibody-induced receptor cross-linking [16]. Hence, a first requirement in order to interrogate the delicate TNFR1/TNFR2 signalling interplay in disease models was the identification and characterisation of a selective inhibitor of mouse TNFR1.

Domain antibodies are single variable domains of full antibodies, contain the structural determinants for antigen recognition, and are one tenth the size of a full mAb [17,18]. They are monomeric and monovalent by design which might be particularly advantageous when targeting TNFR1 given its sensitivity to cross-linking induced agonism. Furthermore, dAbs can be formatted for *in vivo* half-life extension by conjugating to a PEG moiety or by genetic fusion to a second dAb with specific binding to serum albumin. By formatting them for extended half-life, dAbs can be made compatible with dosing regimens in pre-clinical models of chronic disease indications [19].

Therefore, we set out to identify and to characterise anti-mouse TNFR1 dAbs which would selectively inhibit TNFR1 signalling and could be formatted for *in vivo* half-life extension. The primary use of the inhibitor would be as a tool to help understand the delicate balance between TNFR1 and TNFR2 signalling during disease and homeostasis. Furthermore, this tool could help us investigate in pre-clinical disease models what benefits pharmacological inhibition of TNFR1 signalling could provide. During the characterisation of the lead anti-mouse TNFR1 dAb, we discovered a novel mechanism to inhibit TNFR1 signalling alone, without interfering with TNF-α binding to the receptor. We termed this mechanism of action ‘non-competitive’ as no competition with ligand binding was observed. Hence, we report a novel mechanism for TNFR1 inhibition and describe the characterisation of the *in vitro* binding and inhibition properties of a dAb acting through this mechanism. Furthermore, we determined that when formatted as an AlbudAb, the fusion product has pharmacokinetic properties compatible with dosing in chronic disease models and is pharmaco-dynamically active.

## Materials and Methods

### Materials

DMS5540, DMS5538 and DOM1m-21-23 dAb were expressed from a T7 expression vector in *E*. *coli* BL21(*DE3*) strain derivatives. All were purified from culture supernatants by Protein-A (GE Healthcare) affinity capture followed by cation exchange chromatography and polished using size exclusion chromatography when necessary. Final material was concentrated and buffer exchanged into PBS and determined to be >95% purity (SDS-PAGE and SEC) with low endotoxin content. Mouse TNFR2-Fc, a genetic fusion of mouse TNFR2 with mouse IgG2a, was expressed in Chinese Hamster Ovary (CHO1Ea) cells [20] and purified using MabSelectSure and SEC. Recombinant mouse and human TNFR1 chimeras and single point mutants were expressed from pPICZalpha expression vector in *Pichia pastoris* KM71H strain using methanol induction. All TNFR1 mutants were affinity purified from culture supernatants by Ni-NTA agarose (Qiagen) with Imidazole elution. Purified TNFR1 mutants were further Endo H (New England BioLabs; NEB) treated to remove receptor glycosylation with subsequent Endo H removal by Amylose resin (NEB). Recombinant human TNF-α (cat no. 300-1A) was from Peprotech (NJ, USA). Recombinant mouse TNF-α (410-MT/CF), mouse sTNFR1 (425-R1/CF) and human sTNFR1 (636-R1/CF) were from R&D Systems (MN, USA). Relative purity of protein samples were assessed on a non-reducing SDS-PAGE. 12% Bis Tris NuPAGE gels (Invitrogen) were run in MES running buffer as per manufacturer’s protocol. Bands were visualised by staining with InstantBlue (Expedeon). Samples were compared to Novex Sharp pre-stained markers (Invitrogen). Analytical Size Exclusion Chromatography (aSEC) analysis was performed using Agilent 1100 HPLC with TSK G2000 SW_XL_ column at 0.5ml/min flow rate. 10 l of sample was injected using a sodium phosphate pH6.8 based mobile phase containing 5% organic solvent and peaks measured at both 214 and 280 nm wavelength.

Amino acid sequences.

DOM1m-21-23: EVQLLESGGGLVQPGGSLRLSCAASGFTFNRYSMGWLRQAPGKGLEWVSRIDSYGRGTYYEDPVKGRFS ISRDNSKNTLYLQMNSLRAEDTAVYYCAKISQFGSNAFDYWGQGTQVTVSS;

DMS5540:: EVQLLESGGGLVQPGGSLRLSCAASGFTFNRYSMGWLRQAPGKGLEWVSRIDSYGRGTYYEDPVKGRFSISRDNSKNTLYLQMNSLRAEDTAVYYCAKISQFGSNAFDYWGQGTQVTVSSASTDIQMTQSPSSLSASVGDRVTITCRASRPIGTMLSWYQQKPGKAPKLLILFGSRLQSGVPSRFSGSGSGTDFTLTISSLQPEDFATYYCAQAGTHPTTFGQGTKVEIKR;

DMS5538: EVQLLESGGGLVQPGGSLRLSCAASGVNVSHDSMTWVRQAPGKGLEWVSAIRGPNGSTYYADSVKGRFTISRDNSKNTLYLQMNSLRAEDTAVYYCASGARHADTERPPSQQTMPFWGQGTLVTVSSASTDIQMTQSPSSLSASVGDRVTITCRASRPIGTMLSWYQQKPGKAPKLLILFGSRLQSGVPSRFSGSGSGTDFTLTISSLQPEDFATYYCAQAGTHPTTFGQGTKVEIKR

### Mouse fibroblast L929 cell TNF Bioassay

Mouse fibroblast L929 cells (ATCC CCL-1) were seeded (2 x 10^4^/well) in 96-well flat bottom plates and allowed to adhere overnight at 37°C, 5%CO_2_. Cells were washed twice in PBS and a concentration range of test samples and controls were added to the cells and incubated for 1 hour. Then assay media containing Actinomycin D (1.25μg/ml) and mouse TNF-α (final concentration 20pg/ml) were added to the relevant sample and positive control wells and incubated overnight. The following day cell titre96 MTS reagent (Promega, WI, USA) was added to each well and incubated for 1–4 hours before reading at 490nm on a Molecular Devices Spectramax M5e.

### Surface Plasmon Resonance

Biacore experiments were performed on a Biacore T200 instrument in HBS-EP+ buffer (GE healthcare) at 25°C. Biacore CM5 chips (Series S Sensor Chip certified GE Healthcare Bio-Sciences AB, Uppsala, Sweden, BR-1005-30) were coated with either recombinant mouse or human TNFR1 (rhTNFR1) using Amine Coupling Kit according to manufacturer’s instructions (Amine Coupling Kit, GE Healthcare Bio-Sciences AB, Uppsala, Sweden, BR-1000-50). For kinetic experiments, DMS5540 was injected at 7 concentrations, decreasing in 2-fold dilutions from 16 nM to 0.125 nM, over both human and mouse TNFR1 at 50 μl/min. For the TNF-α competition experiment, 1μM recombinant human TNF-α was injected over the chip surface (120s, 20μl/min flow rate). Chip surface was then regenerated back to baseline using regeneration buffer (10mM Glycine, pH 2.0). Next, 1μM DMS5540 was injected and immediately followed by 1μM rhTNF-α (dual injection) or running buffer (HBS-EP+, 10mM HEPES, 150mM NaCl, 3mM EDTA, 0.05% Surfactant P20, pH 7.4). Injection time was 300s for DMS5540. Biacore traces were double referenced and binding responses (in RUs) of rhTNF-α to rhTNFR1 in the absence or presence of DMS5540 were compared. Data collection rate was 10Hz. Association and dissociation parameters were fit using 1:1 binding model in T200 BIAevaluation software.

Competition Biacore experiments used for epitope mapping were performed on a Biacore 3000 instrument in HBS-EP buffer (GE healthcare) at 25°C. Biacore SA chips (SA Sensor Chip GE Healthcare, BR-100032) were coated with either biotinylated recombinant mouse or human TNFR1. Mouse TNFR1 chip surface was confirmed by binding of DOM1m-21-23 (40–80 RU binding depending on chip surface and experiment) compared to reference flow cell and buffer. For competition binding experiments, 50 nM DOM1m-21-23 was incubated 1:1 (v:v) with excess (~30 M) mutant recombinant TNFR1 purified proteins (mouse or human chimera or point mutants) and allowed to reach equilibrium over 1 hour. DOM1m-21-23/TNFR1 mixtures were injected (10 l) over mouse or human TNFR1 chip surfaces at 10 l/min flow rate. Chip surface was then regenerated back to baseline using regeneration buffer (10mM Glycine, pH 2.0). Biacore traces were baseline subtracted and binding responses (in RUs) of DOM1m-21-13 binding to TNFR1 chip surface in the presence and absence of chimeric/mutant mouse or human TNFR1 were compared.

### Mice

#### Ethics statement

All experimental procedures were approved by the Institutional Animal Care and Use Committee at GlaxoSmithKline and by the ethical review process committees at the institution where the work was performed. These committees, the animal handling and the experiments were subject to the UK Home Office and conducted in accordance with the Animals (Scientific Procedures) Act 1986 and the GSK Policy on the Care, Welfare and Treatment of Animals. The murine PK study was performed by Huntingdon Life Sciences (UK) under approved protocol numbers DOMEXP321 /BYL0005. The murine PD study was performed by Biomedcode (Vari, Greece) under approved protocol numbers DOMEXP295/L44412. Mice were housed under standardised light-controlled conditions at room temperature (20±2°C) and 55±15% humidity with free access to food and water. All efforts were made to minimise suffering. For blood sampling mice were either exsanguinated by cardiac puncture under isoflurane/oxygen anaesthesia or sacrificed by CO_2_ administration followed immediately by blood sampling through cardiac puncture.

### Mouse Pharmacokinetic (PK) study

Single intravenous bolus injections, into a caudal vein at dose volumes of 5 mL/kg, of DMS5540 were administered at 0.1, 1.0 and 10 mg/kg to three groups of in total 78 male CD-1 mice (Charles River UK Ltd, Margate, UK). Blood samples, from 3 mice per time point, were taken up to 48 hours (0.1 mg/kg), 96 hours (1 mg/kg) and 216 hours (10 mg/kg). To obtain the blood sample, mice were exsanguinated by cardiac puncture under isoflurane/oxygen anaesthesia. Each blood sample was collected into plain plastic tubes and serum was obtained by centrifugation and frozen for later DMS5540 and sTNFR1 concentration analysis.

### DMS5540 PK bioassay

The concentrations of DMS5540 in mouse PK serum samples were determined using the Meso Scale Discovery (MSD) platform. Briefly, mouse TNFR1-Fc (R&D Systems #430-RI) was coated onto 96-well standard bind MSD plates (MSD #L11XA-6). Wells were then blocked with assay buffer (5% BSA in PBS containing 1% tween-20) and incubated for 1 hour with constant shaking and then washed. Quality control (QC) samples and serum PK samples were added at a range of dilutions alongside a DMS5540 standard curve at a range of known concentrations. Samples and standard curves were incubated for 1 hour at room temperature with constant shaking. Bound DMS5540 was detected with MSD sulfo-tagged rabbit anti-Vκ pAb (in-house reagent). MSD read buffer was added and then the plates were read on a SECTOR 6000 MSD imager. The mean PK profile of DMS5540 in mice after a 0.1mg/kg, 1mg/kg and 10mg/kg *iv* dose was plotted using Graphpad Prism version 4. Derived PK parameters were obtained by plotting mean serum concentrations against time and fitted in WinNonLin (Pharsight version 5.1.1) using non-compartmental modelling. The mean concentrations from each mouse and sparse sampling were used, resulting in a non-compartmental fit of the mean results from 3 mice at each time point.

### sTNFR1 bioassay

The concentration of total soluble TNFR1 in mouse samples containing DMS5540 was determined using a MSD assay. Briefly, 96-well standard bind MSD plates (MSD #L11XA-6) were coated overnight with a rat anti-mouse TNFR1 mAb (R+D Systems #MAB425). Plates were then washed and incubated with assay buffer (PBS containing 3% BSA (Sigma #A7030) and 0.1% Tween-20 (Fisher #BPE337)) followed by incubation with MSD Serum Cytokine Assay Diluent (MSD # R51BB-2). Mouse serum samples were added directly to the relevant wells alongside a standard of mouse sTNFR1 (R&D Systems #425-R1) of known concentrations. Plates were incubated at room temperature for 20 hours before washing. Any bound sTNFR1 was detected using a MSD sulfo-tagged (MSD #R91AN-1) anti-TNFR1 dAb (DMS5541), which recognises an epitope independent of DMS5540. After incubation, plates were washed and 2xMSD read buffer T with surfactant (MSD #R92TC-1) was added and plates were read immediately using the MSD SECTOR 6000.

### Mouse Pharmacodynamic (PD) study

Eight male C57BL6/N mice (at 6 weeks of age; Charles River Laboratories) were dosed in each of the seven groups (56 mice in total) according to the following schedule: T = 0h treatment with concentration range of DMS5540 (4 groups), DMS5538 (1 group) or no dAb (2 groups); T = 4h challenge with 100 ng mouse TNF-α/mouse (6 groups) or no injection (1 group); T = 6h sacrifice all mice for serum IL-6 level determination. The dAbs were prepared and administered intravenously (tail vein) according to the indicated time schedule. The mouse TNF-α challenge consisted of a bolus intravenous (tail vein) administration of 100μl of the 1μg/ml mTNF-α solution. The mice were sacrificed by CO_2_ administration, blood was collected by cardiac puncture and serum was prepared and stored at -80°C. Serum mouse IL-6 levels were determined by ELISA using the Quantikine Mouse IL-6 Immunoassay kit from R&D Systems (LOT:269144, Cat: M6000B), according to the manufacturer’s instructions.

### Statistical analysis

Serum mouse IL-6 levels (pg/ml) were summarised using standard descriptive statistics and graphically presented by each dose group, using boxplots of summary measures and plots of each individual mouse IL-6 serum levels.

To assess and quantify differences in serum mouse IL-6 levels between doses of interest, log-transformed data were analysed using a mixed models repeated measures (MMRM) analysis. Point estimates and 95% CI’s were exponentially back-transformed to obtain adjusted (least square) geometric means for each dose, point estimates and associated 95% CI for the ratio test/reference. Significance tests were performed using a two-sided hypothesis at the 0.05 level, and no adjustment was made for multiple comparisons. Statistical analyses were performed using SAS version 9.1.3 or higher (SAS Institute, Cary, NC, USA).

## Results

### Description of anti-TNFR1 domain antibody

**Fig 1:**
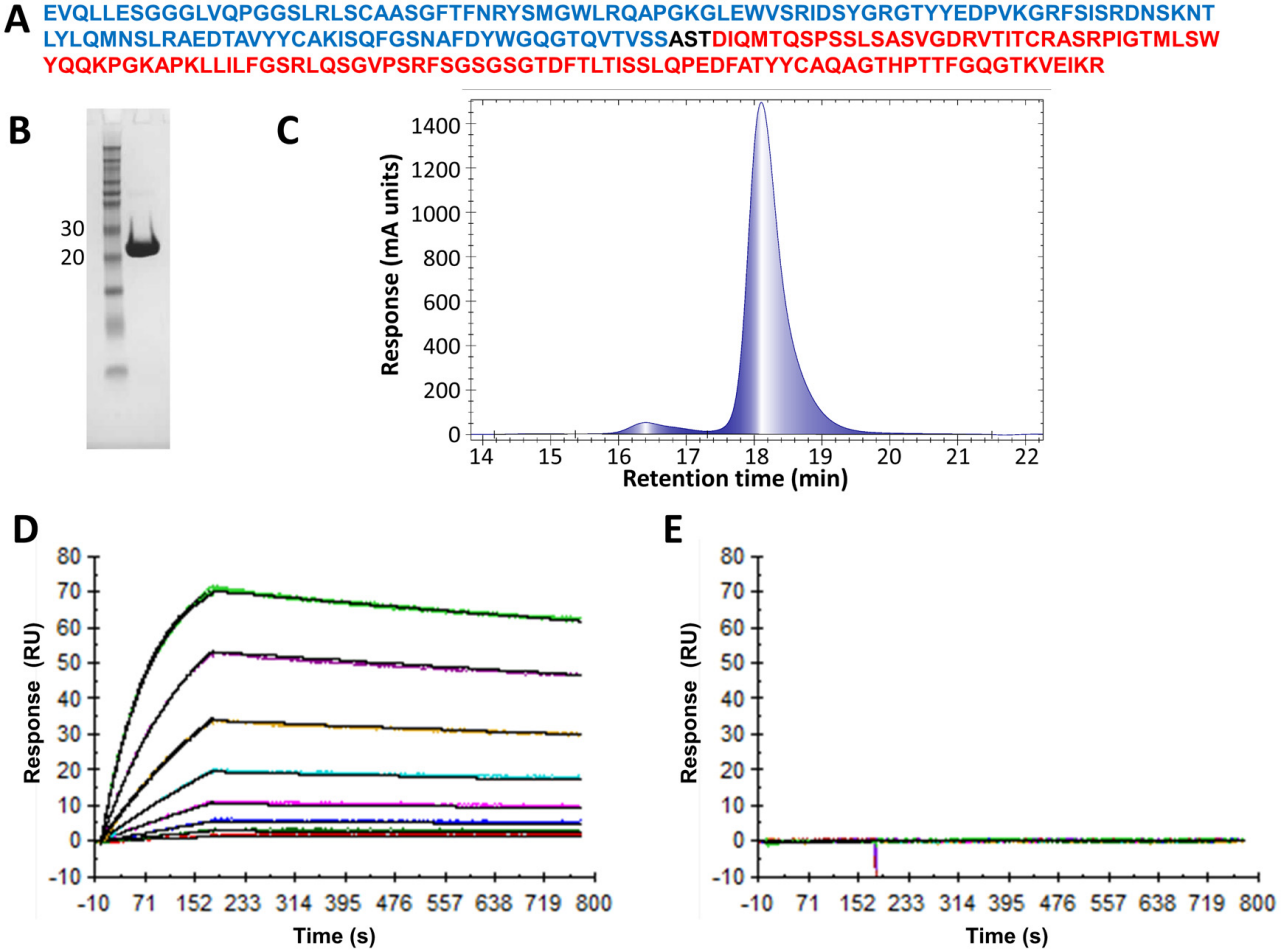
Characterisation of DMS5540 purity and binding properties. (A) Protein sequence of DMS5540: αTNFR1 Vh dAb (blue), short AST linker (black) and Vk AlbudAb (red). (B) SDS-PAGE analysis: 10μg of affinity purified DMS5540 under non-reducing conditions compared with Novex Sharp pre-stained protein markers (Invitrogen). (C) Size exclusion chromatography analysis: Injection (10μl) of 0.6mg/ml DMS5540 onto TSK G2000 SW_XL_ column at 0.5ml/min: main peak at 18.1min (95.5%) that of monomeric DMS5540, with small (<5%) amount of DMS5540 dimer present at 16.4min. Surface plasmon resonance analysis of DMS5540 binding to mouse (D) and human TNFR1 (E). The dissociation equilibrium constants for DMS5540 binding to mouse and human TNFR1 were determined using a Biacore T100. Constants were calculated by injecting DMS5540 over 2-fold dilutions from 16 nM to 0.125 nM over a mouse TNFR1 or human TNFR1 coated chip surface.

An anti-mouse TNFR1-binding dAb was selected from Domantis’ naïve, human dAb phage-display library by biopanning. The binding affinity for mTNFR1 of the selected dAb was further enhanced through a cycle of affinity maturation, using error-prone PCR mutagenesis. The lead dAb identified from this process was named DOM1m-21-23. It was subsequently formatted for *in vivo* half-life extension using the AlbudAb technology in which the anti-mTNFR1 dAb was genetically fused, using a three residue linker (Ala-Ser-Thr), with a mouse albumin binding dAb (DOM7h-11-12) (Fig 1A). The anti-albumin dAb is hypothesised to engage in a reversible interaction with albumin and provide a means to extend the *in vivo* terminal half-life of the anti-TNFR1 dAb. The genetic fusion product (DMS5540) of the two dAbs is about 25kDa in size and was characterised for its *in vitro* and *in vivo* properties. In this study we used DMS5538 as a negative control dAb consisting of a genetic fusion of a VH dummy dAb (VHD2) and the anti-albumin dAb used in DMS5540.

### *In vitro* characterisation of DMS5540 binding and inhibitory activity

DMS5540 was expressed in *E*. *coli* and purified from culture supernatant by Protein-A affinity capture followed by cation exchange chromatography. Product integrity and purity were confirmed by SDS-PAGE analysis (Fig 1B), confirming a product in the 25kDa range, and size exclusion chromatography (Fig 1C). To establish that the fusion product binds mouse TNFR1 with high affinity, we characterised this binding by Surface Plasmon Resonance (Fig 1D). In these Biacore experiments, the mouse TNFR1 was coated on the chip surface and a concentration range of DMS5540 was injected over the surface. The dissociation equilibrium constant (*K*_*D*_) for the DMS5540-mTNFR1 interaction was determined to be 270 pM (*k*_*a*_ 7.98 10^5^ M^-1^s^-1^; *k*_*off*_ 2.17 10^−4^ s^-1^). Binding was specific to mouse TNFR1 as no binding was observed on an adjacent flow cell coated with human TNFR1 (Fig 1E).

**Fig 2:**
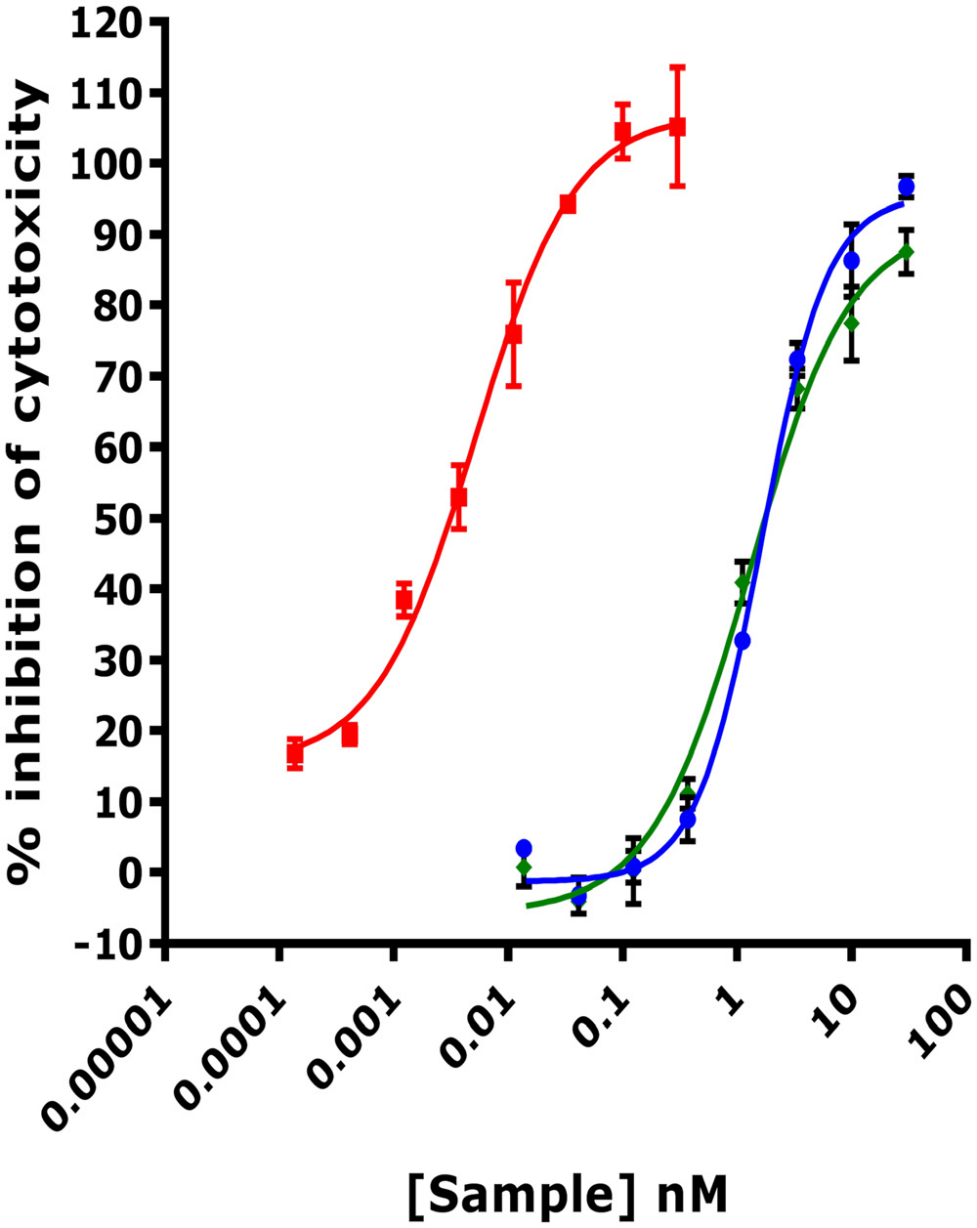
Inhibition of TNF-α induced cytotoxicity in a mouse L929 cell-based assay. DMS5540 (green), DOM1m-21-23 (blue) and mouse TNFR2-Fc (red) were titrated over a 1000-fold concentration range to determine their ability to inhibit mouse TNF-α (20 pg/ml) induced cytotoxicity in L929 cells. Sample concentration is plotted versus % inhibition of cytotoxicity.

The mouse fibroblast cell line L929 is highly sensitive to TNF-α mediated cytotoxicity and therefore frequently used in functional assays to determine the protective effect provided by TNF inhibitors [21]. To determine if DMS5540 was a potent inhibitor of TNF-α induced cytotoxicity, L929 cells were incubated with a concentration range of DMS5540, the anti-mTNFR1 dAb (DOM1m-21-23) or a positive control, mouse TNFR2-Fc (Fig 2). At 20 pg/ml of TNF-α, the anti-TNFR1 molecules both generated inhibition curves resulting in very similar EC50 potency values of 1.3 nM and 1.7 nM for DMS5540 and DOM1m-21-23, respectively. Mouse TNFR2-Fc resulted in an inhibition curve that was nearly 3 orders of magnitude more potent than the TNFR1 inhibitors with an EC50 value of 5pM.

### Non-competitive mechanism of action

**Fig 3:**
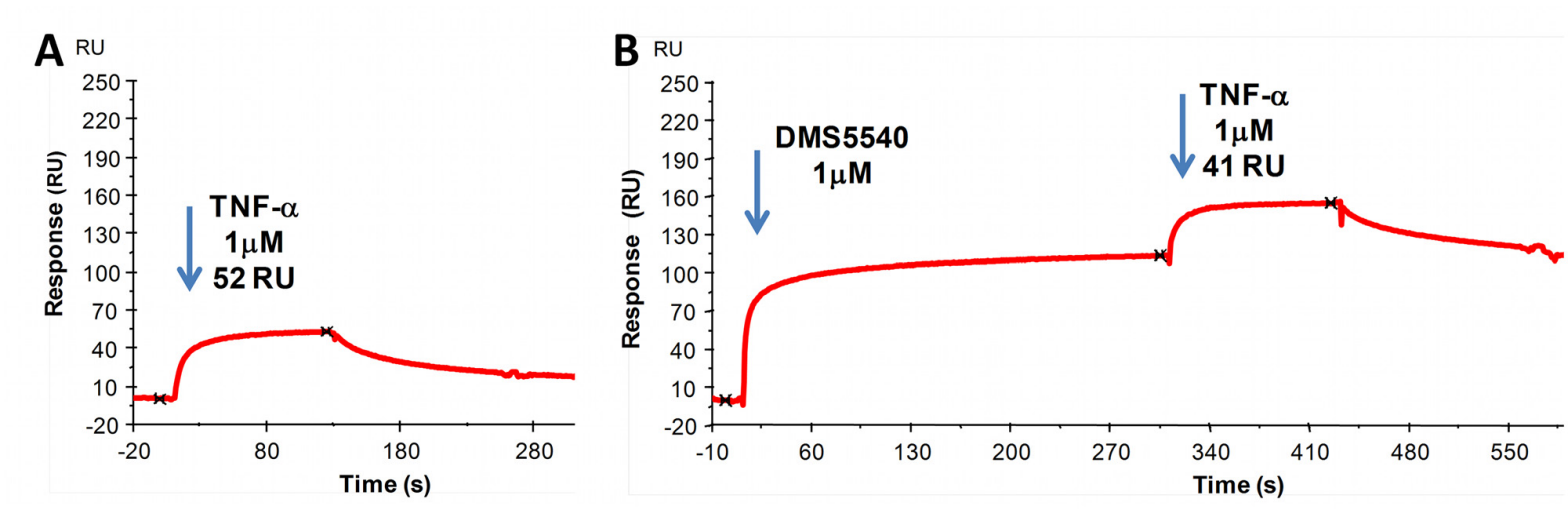
Surface plasmon resonance analysis of simultaneous binding of TNF-α and DMS5540 to mouse TNFR1. (A) Binding of human TNF-α to mouse TNFR1 was established by injecting TNF-α (1 μM) over a Biacore chip surface coated with mouse TNFR1 and quantified as 52RUs. (B) Binding of TNF-α was determined after saturation of chip-bound mouse TNFR1 with DMS5540 and quantified as 41RUs.

To establish if binding of DMS5540 to mouse TNFR1 would interfere in the binding of TNF-α to its receptor, we studied these interactions using Biacore. In these experiments we first established that human TNF-α was able to bind mouse TNFR1 coated on a Biacore chip. An injection of human TNF-α (1 μM) across mouse TNFR1 provided 52RUs of specific binding (Fig 3A). However, after saturating the TNFR1 surface with DMS5540, subsequent injection of human TNF-α (1 μM) still resulted in 41RU binding. Although slightly reduced binding of TNF-α was observed, it would suggest that saturation of TNFR1 with DMS5540 does not preclude binding of TNF-α (Fig 3B). In addition, the shape of the binding curves are very similar, not suggesting any major changes in binding kinetics for TNF-α to TNFR1 in the absence or presence of DMS5540. Therefore, the data would imply that DMS5540 is able to inhibit TNF-α signalling *in vitro*, but does this through a mechanism which, according to our Biacore experiment, does not compete with TNF-α binding to the receptor. We termed this unique mechanism of action ‘non-competitive inhibition of TNFR1’.

### Epitope mapping of anti-TNFR1 dAb

The observation that the anti-TNFR1 dAb, while inhibiting signalling, did not interfere with ligand binding led us to investigate its binding epitope in more detail. Relying on the selective binding of the dAb to mouse TNFR1, but not human TNFR1, we used chimeric receptor constructs in which the first cysteine rich domain (CRD), also known as the pre-ligand assembly domain (PLAD), was exchanged between human and mouse TNFR1. After expression and purification of these receptor constructs, they were tested in a BIAcore competition binding assay with wt mouse TNFR1 for their ability to still be bound by DOM1m-21-23. Whereas the human PLAD domain on mouse CRDs2-4 (HMMM) no longer bound the dAb, the reverse was true for a mouse PLAD on the human CRDs2-4 (MHHH) (Table 1). This identified the PLAD domain to be the dominant domain for binding of the dAb.

**Table 1:** Mapping of DOM1m-21-23 binding epitope on TNFR1.

**A) TNFR1 domain exchange (domains 1234)**					
MMMM	HHHH		HMMM		MHHH
++++	-		-		++++
**B) Fine mapping: mouse TNFR1 domain 1 human substitutions**					
L14V	V21I	V40Y	S41N	R48Q	V51D
++++	++++	++++	++++	++++	-
**C) Human TNFR1 domain 1 reverse substitutions**					
wtD51	D51A		D51T		D51V
-	+		+		++++

The binding epitope of DOM1m-21-23 was determined by characterisation of binding to different mTNFR1 and hTNFR1 mutants using competition Biacore. DOM1m-21-23 was flowed over low density mTNFR1 coated chip surface in the presence or absence of purified receptor variants as indicated. Binding was compared to response units of DOM1m-21-23 binding to mTNFR1 alone. (A) Binding of DOM1m-21-23 to chimeric mutants of TNFR1 where the first CRD was exchanged between mouse (M) and human TNFR1 (H). DOM1m-21-23 binds with equal response to receptors containing mouse domain 1. No competition binding was observed for receptors containing human domain 1. (B) Binding of DOM1m-21-23 to mTNFR1, containing single residue mouse to human substitutions in domain 1, were analysed by competition Biacore as in (A). Binding observed was proportional to mTNFR1 for all substitutions tested with exception of V51D mTNFR1 where no competition was observed. (C) Binding of DOM1m-21-23 to single point substitutions at position D51 on hTNFR1 domain 1 by competition Biacore. Single point substitution hTNFR1 D51V (towards mouse TNFR1 sequence) confers binding to DOM1m-21-23. Low competition binding observed for other variants compared to no binding with that for wild type hTNFR1.

A next step was to fine-map the binding epitope to individual residues by generating full-length, mouse TNFR1 variants with single point reversions to the human amino-acid residues at the positions where these sequences differ in the PLAD domain. A total of 6 different positions (L14V, V21I, V40Y, S41N, R48Q and V51D) were successfully expressed, purified and tested by competition Biacore for continued binding to the dAb. This screen identified V51 in mouse TNFR1 to be required for binding of the dAb. However, as this was a negative screen and loss of binding could be due to a range unrelated causes, e.g. protein misfolding, a positive experiment would be required to confirm this position as the ‘hotspot’ for DOM1m-21-23 binding. To this end, we generated point mutants of full-length human TNFR1 in which D51 was mutated to the corresponding mouse residue (D51V) or related residues (D51T and D51A). Testing of these mutants in a Biacore competition experiment for binding to DOM1m-21-23 demonstrated the ability of a single mutation D51V to rescue binding of DOM1m21-23 to human TNFR1. Hence, binding of the anti-TNFR1 dAb was mapped a single residue in the PLAD domain, which could explain the ability of the dAb to inhibit signalling without inhibiting ligand binding.

### *In vivo* PK studies—target-mediated disposition and effects on total sTNFR1 levels

**Fig 4:**
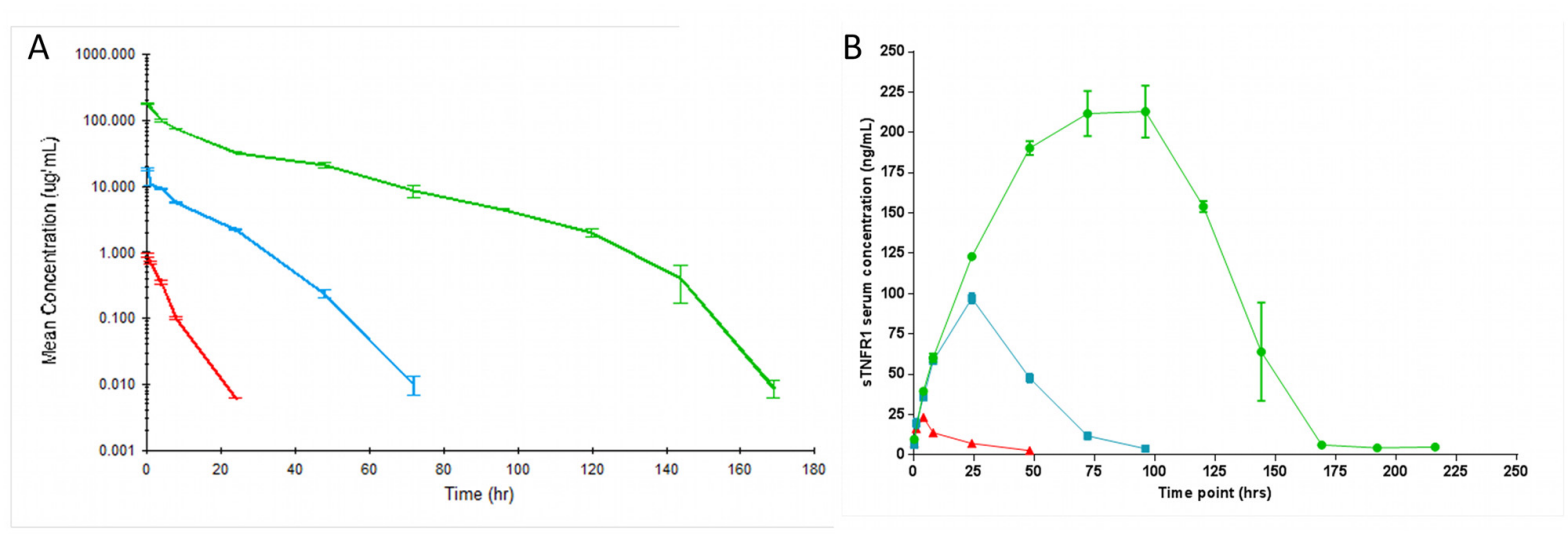
PK and soluble TNFR1 profiles after dosing DMS5540 over a 0.1–10 mg/kg range in mice. (A) Mice were injected *intravenously* with a bolus injection of DMS5540 at 0.1 (red), 1.0 (blue) and 10 mg/kg (green). At each time point, 3 mice were sacrificed and mean DMS5540 serum concentrations (μg/ml) were plotted +/- SEM. (B) Mean (+/- SEM) soluble TNFR1 concentration (ng/ml) over time after injection of 0.1 (red), 1.0 (blue) and 10 mg/kg (green) DMS5540.

To enable the use of DMS5540 in mouse models of chronic disease, the dAb fusion molecule will require a minimum circulating half-life long enough to be compatible with at least daily dosing. We therefore investigated the pharmacokinetic properties of DMS5540 in mice. In addition, by injecting DMS5540 over a concentration range spanning two orders of magnitude (0.1–10 mg/kg), we also investigated whether the kinetics of DMS5540 were non-linear with respect to dose. Mice were dosed intravenous with 0.1, 1 and 10 mg/kg of DMS5540 and the total serum levels of DMS540 were determined over time (Fig 4A). The data indicated that the kinetics were non-linear and that clearance rates decreased as dose of DMS5540 increased. WinNonLin analysis of the data was performed (Table 2) to determine the PK parameters of interest. This analysis determined that the terminal half-life increased from 3.3h after 0.1 mg/kg dosing to 23.2h after the 10 mg/kg dosing. Non-linear PK is most often associated with target-mediated disposition. Hence, the results observed would suggest that DMS5540 is partially cleared through TNFR1-mediated internalisation and that the contribution of this pathway to total clearance is particularly pronounced at lower doses of DMS5540 when serum concentrations are lower and presumably when TNFR1 is not saturated. The observed terminal half-life at the highest dose would imply that in systemic mouse models of disease a dosing frequency of 2–3 times per week would be feasible from a PK perspective.

**Table 2:** PK parameters following dosing with DMS5540 as determined by WinNonLin analysis.

DMS5540 Dose (mg/kg)	T ½ (hr)	Tmax (hr)	Mean Cmax (μg/mL)	SE of Cmax	AUC_(0-∞)_ (hr*μg/mL)	Vz (mL/kg)	Cl (mL/kg/hr)	MRT (hr)
0.1	3.3	0.17	0.9	0.06	4.1	117.9	24.5	3.9
1	8.6	0.17	18.0	0.91	169.8	73.0	5.9	11.5
10	23.2	0.17	178.5	3.35	3028.6	110.6	3.3	27.2

Abbreviations: T1/2: terminal half-life of DMS5540; Tmax: Time at which maximum concentration is reached; Mean Cmax: mean maximum concentration across 3 mice; SE of Cmax: Standard Error of Cmax; AUC: Area Under the concentration/time Curve extrapolated to infinity; Vz: volume of distribution over terminal phase; Cl: Clearance; MRT: Mean Residence Time.

DMS5540 is a genetic fusion of a dAb selected for high-affinity binding to mouse TNFR1 and a second dAb binding serum albumin to extend serum half-life. Consequently, DMS5540 could have the potential to form a ‘bridge’ between soluble TNFR1 (sTNFR1) and serum albumin, leading to a half-life extension of sTNFR1 as well. Furthermore, an increase in sTNFR1 would also confirm binding of DMS5540 to sTNFR1 in the *in vivo* environment. We determined the concentrations of total sTNFR1 and these increased linearly with dose (Fig 4B), implying that DMS5540 bound sTNFR1 in serum and extended the half-life of shed TNFR1. In parallel with DMS5540 clearance, the concentration of total sTNFR1 decreased rapidly and returned to baseline levels as anticipated.

### *In vivo* PD studies

**Fig 5:**
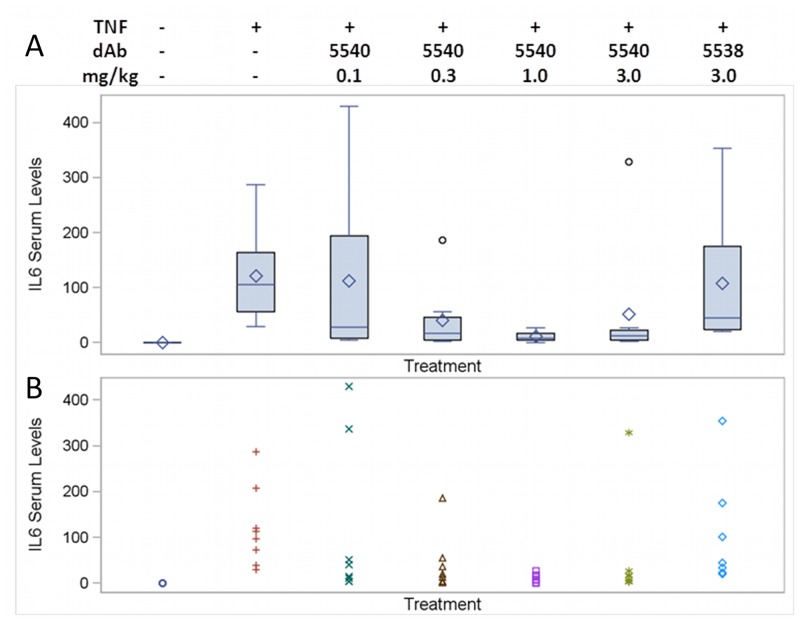
DMS5540 PD determined by protection provided to IL-6 serum increases after *in vivo* TNF-α challenge. Eight mice per dose group were injected with DMS5540, DMS5538 (Dummy control) or nothing at the indicated dose in mg/kg. Four hours later all mice indicated received a bolus injection of mouse TNF-α (100 ng/mouse) and 2 hours later serum samples were taken to determine IL-6 levels. (A) Boxplot for IL-6 serum level (pg/ml) for each dose group. Boxplot description: the horizontal line is the median and the diamond is the mean. The upper and lower ends of the box are located at the upper quartile (Q3) and lower quartile (Q1) respectively. The whiskers show the minimum observation before 1.5 x IQR below the box and the maximum observation before 1.5 x IQR above the box. Observations beyond the error bars are shown as individual data points. (B) Individual mouse IL-6 serum levels (pg/ml) for all 8 mice in each dose group.

Finally, we set out to investigate the pharmacodynamic properties of DMS5540 in an *in vivo* challenge study. The challenge model consisted of DMS5540-dosed mice receiving an *intravenous* bolus injection of mouse TNF-α and determining the effects on serum IL-6 levels. The chosen TNF-α dose (100 ng/25g mouse) generated a robust IL-6 response without leading to any visible discomfort to the mice or any behavioural changes that would indicate ethical concerns for the study to progress. In these studies, eight mice per dose group were first injected intravenously with i) a range of DMS5540 concentrations (0.1–3.0 mg/kg), ii) the negative control dAb (DMS5538) (3 mg/kg) or iii) no dAb. Four hours later, all these mice received mouse TNF-α (100ng/mouse). Serum samples were taken 2 hrs after the TNF-α challenge and the serum levels of IL-6 were determined. The IL-6 response data are summarised in boxplots for each dose group in Fig 5A and shown for each individual mouse in Fig 5B. To determine whether the observed differences were significant, a statistical analysis was done in which the adjusted (least square) geometric means for the different treatment groups were compared and the 95% Confidence Intervals (CI) were calculated (Table 3). From the 0.3 mg/kg dose of DMS5540 upwards, the observed decrease in IL-6 response becomes significant compared to the TNF-α only group (p = 0.02). For the 1 mg/kg dose group, IL-6 levels were inhibited by >95%, which was highly significant compared to either the TNF-α only or negative control dAb (DMS5538) group, p = 0.0001 or p = 0.001, respectively. The differences observed for the 3 mg/kg dose group continued to be significant compared to both control groups. Hence, DMS5540 demonstrated the ability to inhibit TNF-α-mediated signalling effects *in vivo* and could therefore be an appropriate tool reagent to investigate the effects of selective, pharmacological inhibition of mouse TNFR1 in pre-clinical disease models.

**Table 3:** Statistical analysis comparing effects of dAb dosing on IL-6 serum levels after a TNF-α challenge.

Comparison (mg/kg)	Adjusted Geometric Means (IL-6/pg/ml)		Ratio (Test/Ref)	95% CI^#^	P-Value
	Test	Reference TNF or 5538			
5540 (0.1) vs TNF Only	31.07	95.16	0.33	(0.07, 1.47)	0.1419
5540 (0.3) vs TNF Only	16.46	95.16	0.17	(0.04, 0.78)	0.0231*
5540 (1.0) vs TNF Only	4.1	95.16	0.04	(0.01, 0.19)	0.0001*
5540 (3.0) vs TNF Only	13.38	95.16	0.14	(0.03, 0.63)	0.0116*
5538 (3.0) vs TNF Only	64.17	95.16	0.67	(0.14, 3.20)	0.6143
5540 (0.1) vs 5538 (3.0)	31.07	64.17	0.48	(0.10, 2.30)	0.355
5540 (0.3) vs 5538 (3.0)	16.46	64.17	0.26	(0.05, 1.22)	0.086
5540 (1.0) vs 5538 (3.0)	4.1	64.17	0.06	(0.01, 0.30)	0.001*
5540 (3.0) vs 5538 (3.0)	13.38	64.17	0.21	(0.04, 0.99)	0.049*

* Statistically Significant

^#^ The point estimate provides the best estimate of the true ratio. The 95% confidence interval provides a range of plausible values for the ratio or difference.

The adjusted geometric means in IL-6 serum concentration when dosing DMS5540 (5540) have been compared against either dosing of TNF-α only or dosing of Dummy control DMS5538 (5538) at 3 mg/kg. The quantities of dAb dosed are indicated in mg/kg and 95% Confidence Intervals (CI) were calculated based on the eight mice dosed per group. Details of the statistical analysis are described in Material and Methods.

## Discussion

Here we describe the characterisation of a monovalent antagonist of mouse TNFR1. The antagonist binds mouse TNFR1 with high affinity, but not human TNFR1, and inhibits TNF-α-mediated cytotoxicity in the L929 cell-based assay. Surprisingly, the dAb does not inhibit TNF-α binding to its receptor, as determined by Biacore, which was supported by data showing its binding epitope mapped to the PLAD domain of TNFR1. Because of this unique mechanism of action, we termed the inhibitor a non-competitive inhibitor of TNFR1.

Structurally, the extracellular portion of TNFR1 consists of four cysteine-rich domains (CRDs). CRD1 has also been described as the pre-ligand assembly domain (PLAD) [22]. Disruption of PLAD domain interactions has been reported to inhibit TNFR1 signalling and to be beneficial in models of arthritis [23]. CRD2 and CRD3 are involved in ligand binding [24] and CRD4 is located closest to the membrane and in the vicinity of the site at which TNFR1 is shed for release in its soluble form. From our Biacore experiments showing that DMS5540 does not interfere with TNF-α binding to TNFR1, we would conclude that the binding epitope for DMS5540 is not in CRD2 or 3. Furthermore, the observation that *in vivo* sTNFR1 concentrations increased proportionally to the increase in DMS5540 dose, would suggest that DMS5540 does not inhibit the shedding rate of TNFR1 and therefore might not be binding in CRD4. This would lead us to hypothesise that the most likely binding epitope for DMS5540 could be in CRD1/PLAD, as binding in this region might accomplish inhibition of TNFR1 signalling without interfering with ligand binding or receptor shedding. Our epitope mapping studies, using the selectivity of the anti-TNFR1 dAb for mouse but not human TNFR1, confirmed the binding epitope to be in the PLAD and to consist of a single ‘hotspot’ residue V51 in mouse TNFR1. Our *in vitro* and *in vivo* data therefore suggests that TNFR1 signalling can be inhibited by the non-competitive mechanism of action of DMS5540.

The primary aim of the PK study was to confirm that by genetically fusing the anti-TNFR1 dAb with an AlbudAb we can extend the terminal half-life of the antagonist to make it compatible with dosing frequencies required for pre-clinical disease models. Monomeric dAbs, in the absence of a half-life extension technology, have been described to have a terminal half-life in mouse serum of about 42min [19]. The ca. 30-fold increase in terminal half-life (23.2 hrs) observed for DMS5540 confirms the ability of the AlbudAb platform technology to extend the half-life of short-lived molecules. Furthermore, it provides confidence that a convenient dosing frequency can be achieved in pre-clinical models. The second observation made from the PK study is the non-linear clearance of DMS5540 over a range of dosing concentrations. Target-mediated disposition is frequently observed when targeting proteins with a significant internalisation rate, *e*.*g*. receptor targets, and is associated with non-linear clearance kinetics [25]. The increased clearance of DMS5540 at lower doses is therefore suggestive of TNFR1 interaction and the rates might be used to calculate receptor turn-over constants. Further evidence of *in vivo* target interaction can be found in the dose dependent increases in total sTNFR1 levels as determined over time. An interesting possibility might be that due to the non-competitive mechanism of DMS5540, the increased sTNFR1 might still be able to bind TNF-α and inhibit its activity. However, as can be seen in Fig 3A, the dissociation equilibrium rate constant of sTNFR1 for TNF-α is weak. This would suggest that concentrations of sTNFR1 would need to be higher than achieved at our highest DMS5540 dose to inhibit effectively TNF-α activity *in vivo*.

The ability of DMS5540 to inhibit TNF-α-mediated cytotoxicity in an *in vitro* L929 cell-based assay does not guarantee *in vivo* functional activity. Therefore, we performed the TNF challenge model and used the IL-6 cytokine read-out as a measure for TNFR1 inhibition by DMS5540. The dose-dependent decrease in IL-6 serum levels confirms that DMS5540 is a functional inhibitor of TNFR1 signalling *in vivo*. In addition, the reduction of >95% in IL-6 serum levels implies i) that a non-competitive anti-TNFR1 dAb is able to achieve near complete inhibition of TNFR1 signalling *in vivo*, ii) that binding to serum albumin does not limit the *in vivo* potential of DMS5540 to inhibit TNFR1 signalling, and iii) this inhibition is achieved at serum TNF-α peak concentrations (~50 ng/ml) in vast excess of those present in either homeostasis (1–10 pg/ml) or disease conditions (10–500 pg/ml). These data complement our previously reported use of DMS5540 in a mouse collagen-induced arthritis model, where it was able to inhibit disease progression similar to an anti-TNF [26]. Hence, DMS5540 could be an appropriate tool reagent for *in vivo* pre-clinical mouse experiments to dissect the role and potential benefits of selective TNFR1 inhibition.

Currently available reagents to dissect TNFR1/TNFR2 biology in mice are the dominant negative TNF muteins, e.g. R1antTNF [27] or XPro1595 [28], and antagonistic monoclonal antibodies. The muteins function by intercalating with wt TNF and rendering it unable to bind TNFR1 while maintaining TNFR2 binding. The small size and short half-life of TNF-α in vivo require PEGylation of the muteins prior to use in in vivo mouse experiments. Furthermore, as the muteins target the soluble ligand and do not inhibit membrane TNF, they have also been used to investigate the contributions of membrane versus soluble TNF activity [28]. Consequently, it might not always be clear if observed effects are due to TNFR2 or to a contribution of membrane bound TNF. Although we have no data to demonstrate that DMS5540 specifically inhibits membrane TNF, it would be a reasonable assumption to make as the inhibitory effect of the dAb is receptor mediated. The main anti-mouse TNFR1 antagonistic, monoclonal antibody described is a hamster anti-mouse (55R-170) [29]. Although use of this antibody has been described in a LPS-induced model [30], detailed dose response in the absence of ligand ruling out any agonism activity at low dose have not been described. In addition, the antibody is of hamster origin and contains an IgG Fc-region, which might mediate additional activities beyond only inhibiting TNFR1 activity. DMS5540 lacks any Fc-region mediated activity as it only consists of two human single variable domains fused through a genetic linker. Hence, DMS5540 might provide a valuable expansion to the existing repertoire of selective mouse TNFR1 antagonists.

Using our selective mouse TNFR1 inhibitor in the collagen-induced arthritis model, we demonstrated different effects when inhibiting TNFR1 compared to pan-TNF-α inhibition with mouse TNFR2-Fc. Specifically, the observed impact of DMS5540 treatment on the number of Treg cells and their level of suppressive activity could be potentially beneficial. Additional disease indications have been described where inhibition of inflammatory TNFR1 activity, while maintaining TNFR2-associated Treg suppressive activity, might be beneficial, *e*.*g*. type-1 diabetes [8], heart failure [31] and multiple sclerosis [32]. DMS5540 could provide a very useful tool in mouse models of these disease indications to dissect the individual contributions of TNFR1/R2 signalling and establish if a potential clinical benefit in these indications could exist for selective receptor targeting.
